# Investigating the Cardiovascular Benefits of Dapagliflozin: Vasodilatory Effect on Isolated Rat Coronary Arteries

**DOI:** 10.3390/ijms242316873

**Published:** 2023-11-28

**Authors:** Sooyeon Choi, Chae Eun Haam, Seonhee Byeon, Eun Yi Oh, Soo-Kyoung Choi, Young-Ho Lee

**Affiliations:** Department of Physiology, Yonsei University College of Medicine, 50 Yonseiro, Seodaemun-gu, Seoul 03722, Republic of Korea; doh0902@yonsei.ac.kr (S.C.); cehaam@yonsei.ac.kr (C.E.H.);

**Keywords:** SGLT2 inhibitor, dapagliflozin, coronary artery, relaxation

## Abstract

Dapagliflozin, a sodium–glucose co-transporter 2 (SGLT2) inhibitor, is an antidiabetic medication that reduces blood glucose. Although it is well known that dapagliflozin has additional benefits beyond glycemic control, such as reducing blood pressure and lowering the risk of cardiovascular events, no sufficient research data are available on the direct effect of dapagliflozin on cardiovascular function. Thus, in this study, we investigated the direct vascular effect of dapagliflozin on isolated rat coronary arteries. The left descending coronary arteries of 13-week-old male Sprague Dawley rats were cut into segments 2–3 mm long and mounted in a multi-wire myography system to measure isometric tension. Dapagliflozin effectively reduced blood vessel constriction induced by U-46619 (500 nM) in coronary arteries regardless of the endothelium. Treatment with an eNOS inhibitor (L-NNA, 100 μM), sGC inhibitor (ODQ, 5 μM), or COX inhibitor (indomethacin, 3 μM) did not affect the vasodilation induced by dapagliflozin. The application of a Ca^2+^-activated K^+^ channel (K_Ca_) blocker (TEA, 2 mM), voltage-dependent K^+^ channel (K_V_) blocker (4-AP, 2 mM), ATP-sensitive K^+^ channel blocker (K_ATP_) glibenclamide (3 μM), and inward-rectifier K^+^ channel (K_IR_) blocker (BaCl_2_, 30 μM) did not affect the dapagliflozin-induced vasodilation either. The treatment with dapagliflozin decreased contractile responses induced by the addition of Ca^2+^, which suggested that the extracellular Ca^2+^ influx was inhibited by dapagliflozin. Treatment with dapagliflozin decreased the phosphorylation level of the 20 kDa myosin light chain (MLC_20_) in vascular smooth muscle cells. In the present study, we found that dapagliflozin has a significant vasodilatory effect on rat coronary arteries. Our findings suggest a novel pharmacologic approach for the treatment of cardiovascular diseases in diabetic patients through the modulation of Ca^2+^ homeostasis via dapagliflozin administration.

## 1. Introduction

Type 2 diabetes (T2D) is characterized by a chronic elevation of blood sugar levels due to insulin resistance and impaired insulin secretion, resulting in disturbances in the regulation of carbohydrate, lipid, and protein metabolism [1]. Type 2 diabetes is a prevalent global health issue, accounting for nearly 90% of approximately 530 million cases of diabetes worldwide [2]. In addition to its well-known defects in blood sugar control, T2D also involves an increased risk of various cardiovascular complications, including coronary artery disease, heart failure, stroke, and peripheral artery disease [3]. Cardiovascular diseases (CVDs) remain leading causes of morbidity and mortality in individuals with T2D, emphasizing the need for comprehensive treatment strategies that address both glycemic control and cardiovascular risk factors [4].

**Figure 1:**
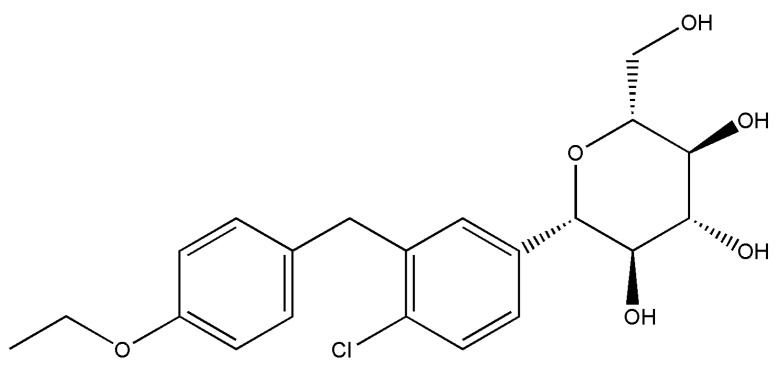
Chemical structure of dapagliflozin.

In recent years, a new class of antidiabetic medications, sodium–glucose cotransporter 2 (SGLT2) inhibitors, has emerged with the potential to offer cardiovascular benefits beyond glucose management. To date, the U.S. Food and Drug Administration (FDA) has approved the use of various SGLT2 inhibitors, including canagliflozin, dapagliflozin, and empagliflozin, in patients with T2D [5]. Among these inhibitors, dapagliflozin stands out as a promising member of this class. Dapagliflozin is chemically described as (2S,3R,4R,5S,6R)-2-[4-chloro-3-[(4-ethoxyphenyl)methyl]phenyl]-6-(hydroxymethyl)oxane-3,4,5-triol (Figure 1). The molecular weight of dapagliflozin is 408.87 g/mol, and its molecular formula is C_21_H_25_ClO_6_ [6].

Dapagliflozin’s primary mode of action involves inhibiting glucose reabsorption in the kidneys, leading to increased urinary glucose excretion and improved glycemic control [7]. Beyond its glucose-lowering effects, dapagliflozin has garnered significant attention for its intriguing cardiovascular effects, making it a subject of interest for both basic researchers and clinicians.

Various clinical trials, including the DECLARE-TIMI 58 (Dapagliflozin Effect on Cardiovascular Events) and Dapa-HF (dapagliflozin and prevention of adverse outcome in heart failure) studies, have revealed the potential cardioprotective effects of dapagliflozin for patients with T2D [8]. These trials demonstrated that dapagliflozin treatment significantly reduced major adverse cardiovascular events, including cardiovascular death, non-fatal myocardial infarction, and stroke [4,9]. Consequently, an increasing number of studies are being conducted to explore the underlying mechanisms explaining the observed cardioprotective effects of dapagliflozin. Recent research suggested that treatment with dapagliflozin can improve endothelial function, reduce arterial stiffness, and exhibit anti-inflammatory effects in patients with T2D [10]. These multifaceted cardiovascular benefits position dapagliflozin as a potential adjunctive therapy to alleviate the burden of CVD in individuals with T2D. Although there is evidence to support the cardiovascular benefits of dapagliflozin, further research is needed to gain a deeper understanding of the underlying mechanisms [11]. Thus, it is necessary to elucidate the direct effect of dapagliflozin on blood vessels, which contributes to understanding the pleiotropic effects of dapagliflozin. Therefore, this study aims to investigate the direct effect of dapagliflozin on the cardiovascular system, with a specific focus on vascular function in coronary arteries.

## 2. Results

### 2.1. Effect of Dapagliflozin on High-K^+^- or U-46619-Induced Contraction in Rat Coronary Arteries

**Figure 2:**
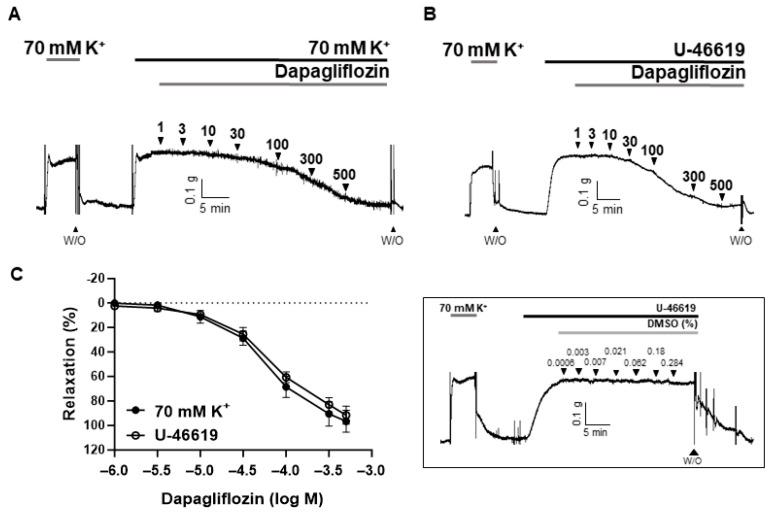
Dapagliflozin-induced vasodilation in rat coronary arteries. Representative traces showing response to cumulative administration of dapagliflozin (1–500 μM) on high K^+^- (**A**) or U-46619 (500 nM)-induced contraction (**B**). Statistical analysis of the relaxation response to dapagliflozin (**C**). Mean ± SD (*n* = 6). Inset: representative trace showing responses to vehicle, DMSO (0.0006–0.284%). (W/O: wash out).

To investigate the effect of dapagliflozin on vascular function, we conducted a cumulative administration of dapagliflozin on coronary arteries pre-constricted with high K^+^ (70 mM) or U-46619 (500 nM). Dapagliflozin (1–500 μM) demonstrated a concentration-dependent vasodilation in the coronary arteries (Figure 2A,B). The dapagliflozin-induced vasodilation was 94.91 ± 7.05% in arteries constricted with high K^+^ and 92.41 ± 6.80% in arteries constricted with U-46619. The effective concentration of dapagliflozin at 50% of its maximum vasodilation (EC_50_) was determined to be 70.2 μM.

### 2.2. Effects of SGLT2 Inhibition on Rat Coronary Arteries

**Figure 3:**
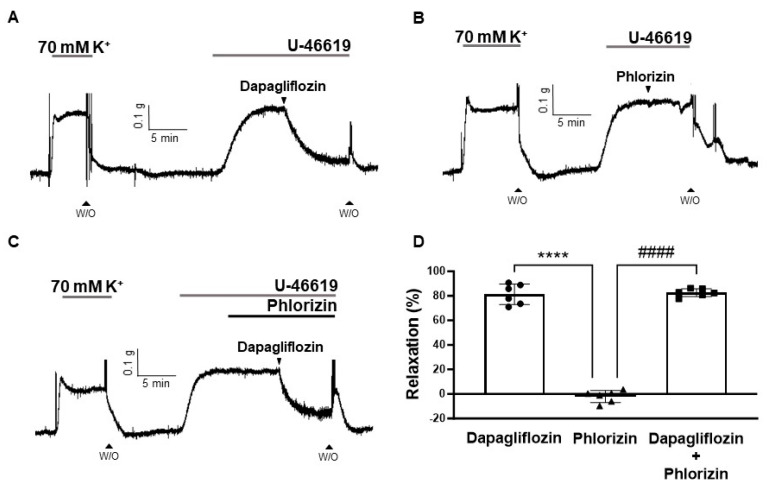
Comparison of vasorelaxant effects induced by dapagliflozin and phlorizin. Original traces illustrating vasodilation induced by dapagliflozin (500 μM, (**A**)), phlorizin (10 μM, (**B**)), and a combination of dapagliflozin (500 μM) and phlorizin (10 μM, (**C**)). Statistical analysis of relaxation responses (**D**). Mean ± SD (*n* = 6). **** *p* < 0.0001, dapagliflozin vs. phlorizin. ^####^ *p* < 0.0001, dapagliflozin + phlorizin vs. phlorizin. (W/O: wash out).

To investigate whether dapagliflozin-induced vasodilation could be attributed to SGLT inhibition, we applied phlorizin, a non-selective inhibitor of SGLT1 and SGLT2, to coronary arteries and analyzed its vasodilatory response. Our data showed that dapagliflozin (500 μM) caused a degree of relaxation of 81.25 ± 8.25% in U-46619-contracted coronary arteries, whereas phlorizin (10 μM) exhibited no vasodilatory effect (−2.06 ± 4.93%, Figure 3B). The administration of dapagliflozin (500 μM) in combination with phlorizin (10 μM) resulted in substantial vasodilation (82.57 ± 3.19, Figure 3C), which is similar to the degree of relaxation induced by dapagliflozin (500 μM) alone (81.25 ± 8.25%).

### 2.3. Dapagliflozin-Induced Endothelium-Independent Relaxation

**Figure 4:**
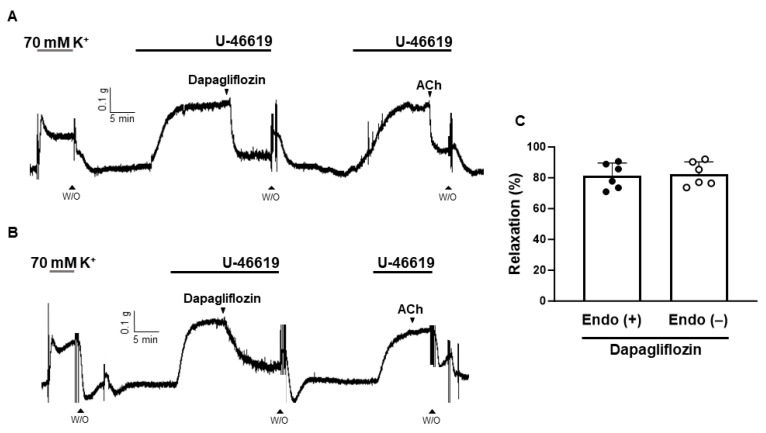
Endothelium-independent vasodilation induced by dapagliflozin. Representative traces showing dapagliflozin (500 μM)-induced vasodilation in the endothelium-intact (**A**) and endothelium-denuded coronary arteries (**B**). Statistical of dapagliflozin-induced vasodilation (**C**). Mean ± SD (*n* = 6). (ACh: acetylcholine; W/O: wash out).

To determine whether the vasodilation induced by dapagliflozin is dependent on the presence of the endothelium, we applied dapagliflozin to endothelium-intact (Figure 4A) and endothelium-denuded coronary arteries (Figure 4B). The maximal value of dapagliflozin-induced vasodilation was 81.25 ± 8.25% in the endothelium-intact arteries and 82.50 ± 7.78% in the endothelium-denuded arteries. There was no significant difference in the vasodilatory effects of dapagliflozin between endothelium-intact and endothelium-denuded coronary arteries (Figure 4C). 

### 2.4. Effects of L-NNA, ODQ, and Indomethacin on Dapagliflozin-Induced Vasodilation

**Figure 5:**
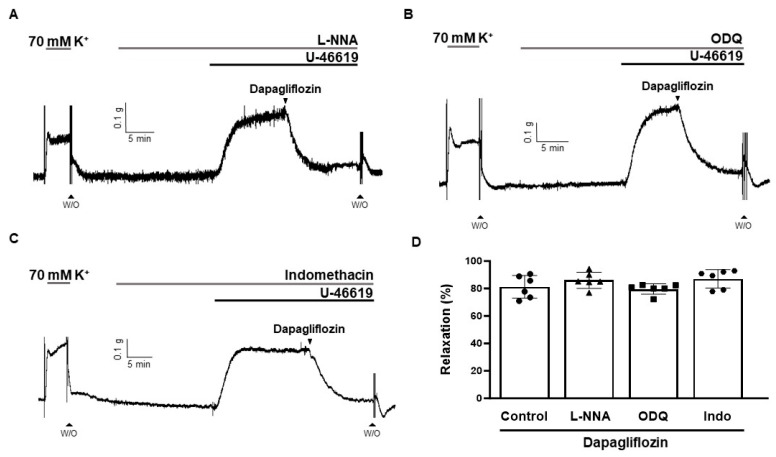
Effects of L-NNA, ODQ, and indomethacin on dapagliflozin-induced vasodilation. Representative traces showing dapagliflozin (500 μM)-induced vasodilation in the presence of L-NNA (100 μM, (**A**)), ODQ (5 μM, (**B**)), and indomethacin (3 μM, (**C**)). Statistical analysis of the relaxation response of dapagliflozin in the presence of L-NNA, ODQ, and indomethacin (**D**). Relaxation of arteries is expressed as the percentage of the contraction induced by U-46619 (500 nM). Mean ± SD (*n* = 6). (L-NNA: *N*^ω^-Nitro-l-arginine; ODQ: 1H-[1,2,4]-oxadiazolo-[4,3-α]-quinoxalin-1-one; W/O: wash out).

To confirm that dapagliflozin-induced vasodilation is endothelium-independent, and to further investigate the involvement of the nitric oxide (NO)/cyclic guanosine monophosphate (cGMP) pathways and cyclooxygenase (COX)/prostacyclin (PGI_2_), the arteries were exposed to a 20 min incubation period with an endothelial nitric oxide synthase (eNOS) inhibitor (*N*^ω^-Nitro-l-arginine (L-NNA, 100 µM)), a soluble guanylyl cyclase (sGC) inhibitor (1*H*-(1,2,4)oxadiazolo[4,3-*a*]quinoxalin-1-one (ODQ, 5 µM)), or a COX inhibitor (indomethacin, 3 µM), prior to contraction with U-46619 (500 nM). The vasodilatory responses induced by dapagliflozin were 86.00 ± 5.84%, 79.64 ± 3.80%, and 87.76 ± 7.94%, in the presence of L-NNA, ODQ, and indomethacin, respectively (Figure 5).

### 2.5. Effects of K^+^ Channel Blockers on Dapagliflozin-Induced Vascular Relaxation

**Figure 6:**
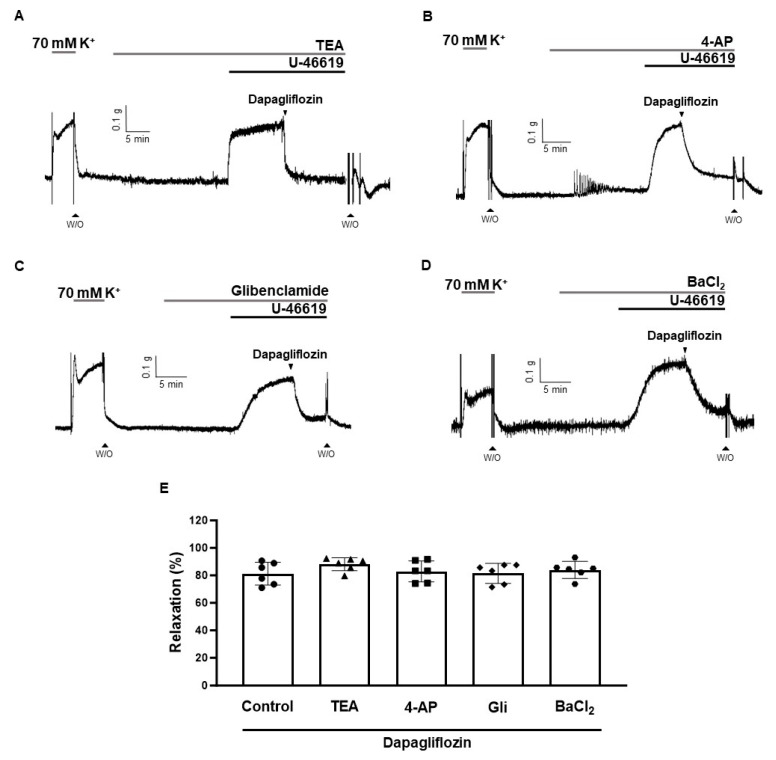
Effects of K^+^ channel blockers on dapagliflozin-induced vasodilation. Effects of dapagliflozin (500 μM) in coronary arteries pre-contracted with U-46619 (500 nM) in the presence of TEA (2 mM, (**A**)), 4-AP (2 mM, (**B**)), glibenclamide (3 μM, (**C**)), or BaCl_2_ (30 μM, (**D**)). Statistical analysis of the relaxation response of dapagliflozin in the presence of K^+^ blockers. Relaxation of arteries is expressed as the percentage of the contraction induced by U-46619 (**E**). Mean ± SD (*n* = 6). (TEA: tetraethylammonium; 4-AP: 4-aminopyridine; Gli: glibenclamide; W/O: wash out).

To determine whether K^+^ channels are involved in the vasodilatory response induced by dapagliflozin, we pre-treated the arteries with various K^+^ channel blockers: a non-selective K^+^ channel blocker, tetraethylammonium chloride (TEA, 2 mM); a voltage-dependent K^+^ channel blocker, 4-aminopyridine (4-AP, 2 mM); an ATP-sensitive K^+^ channel blocker, glibenclamide (3 μM); and an inward rectifier K^+^ channel blocker, barium chloride (BaCl_2,_ 30 μM). These blockers were administered 20 min prior to treatment with U-46619 (500 nM). The relaxation responses of dapagliflozin were 88.12 ± 4.77%, 82.78 ± 7.88%, 81.47 ± 7.23%, and 84.35 ± 8.04%, in the presence of TEA, 4-AP, glibenclamide, and BaCl_2_, respectively (Figure 6). Treatment with K^+^ channel blockers did not affect the relaxation response induced by dapagliflozin.

### 2.6. Effect of Dapagliflozin on Extracellular-Ca^2+^-Induced Contraction

**Figure 7:**
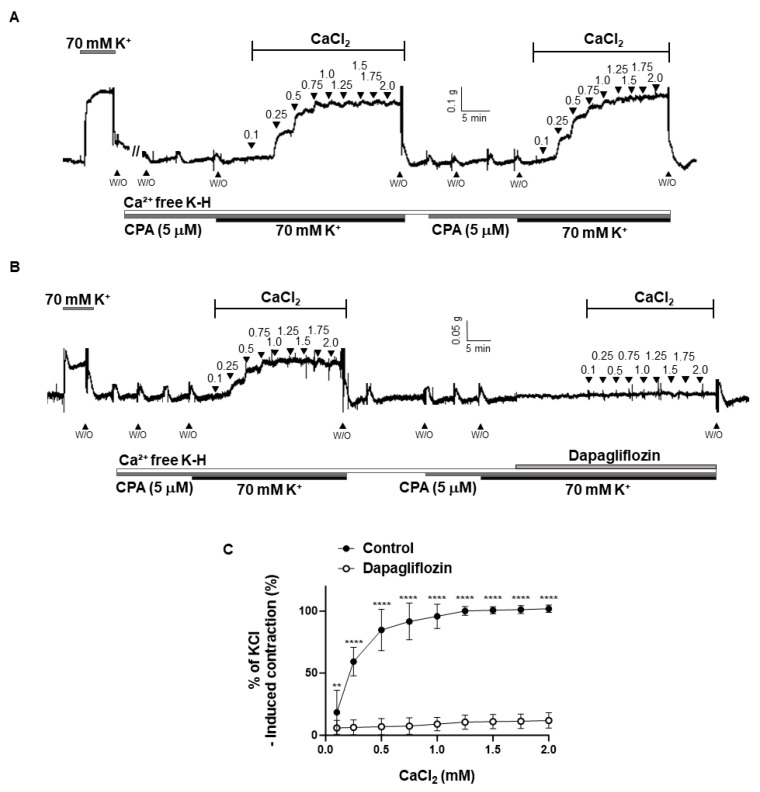
Inhibition of extracellular-Ca^2+^-induced vasoconstriction by dapagliflozin. Representative traces showing the contraction responses to the repeated addition of Ca^2+^ (0.1–2.0 mM) in the absence of dapagliflozin (**A**) and in the presence of dapagliflozin (500 μM, (**B**)). Statistical analysis of the contraction induced by CaCl_2_ in the coronary arteries with or without dapagliflozin (**C**). Mean ± SD (*n* = 6). ** *p* < 0.01, **** *p* < 0.0001, control vs. dapagliflozin. (W/O: wash out; CPA: cyclopiazonic acid).

To investigate whether dapagliflozin’s vasodilatory effects are associated with the inhibition of extracellular Ca^2+^ influx, we examined the effect of dapagliflozin on contractile responses induced via the cumulative addition of CaCl_2_ (0.1–2.0 mM). These responses were observed in arteries that had been incubated in a Ca^2+^-free K-H solution containing a sarcoplasmic reticulum Ca^2+^-ATPase (SERCA) inhibitor, cyclopiazonic acid (CPA, 5 µM), and high K^+^ (70 mM). It was confirmed that the contractile responses elicited by the repetitive addition of CaCl_2_ remained unaffected (Figure 7A). Pre-treatment with dapagliflozin (500 μM) significantly decreased the contractile responses elicited by the cumulative addition of CaCl_2_ (Figure 7B,C).

### 2.7. Decrease in Phosphorylation Level of MLC_20_ by Dapagliflozin in Human Aortic Smooth Muscle Cells

**Figure 8:**
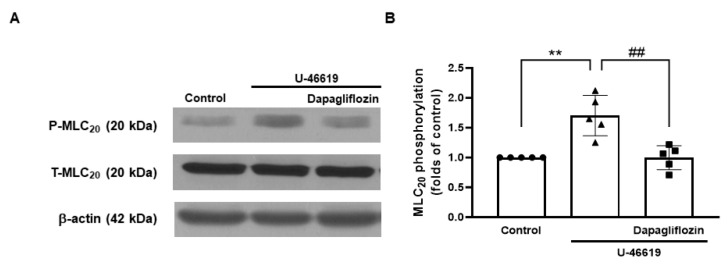
Effect of dapagliflozin on the phosphorylation of 20 kDa myosin light chain (MLC_20_). Representative Western blot analysis for phosphorylated MLC_20_ (P-MLC_20_) and total MLC_20_ (T-MLC_20_) in control, AoSMCs treated with U-46619 (100 nM), and AoSMCs co-treated with U-46619 and dapagliflozin (50 μM, (**A**)). Quantitative data for phosphorylated MLC_20_ (**B**). Mean ± SD (*n* = 5). ** *p* < 0.01; control vs. U-46619; ^##^ *p* < 0.01; control vs. U-46619 + dapagliflozin.

To investigate whether the relaxation induced by dapagliflozin resulted from reduced MLC_20_ phosphorylation, we assessed the phosphorylation and total expression level of MLC_20_ in human aortic smooth muscle cells. (AoSMCs, Figure 8). The administration of U-46619 (100 nM) increased the phosphorylation level of MLC_20_ in AoSMCs, which was significantly reduced by the treatment with dapagliflozin (50 μM).

## 3. Discussion

Recent clinical trials reported that dapagliflozin treatment can reduce the risk of death from cardiovascular disease by improving glycemic and blood pressure control [8,12,13]. However, the exact mechanism of the blood-pressure-lowering effect of dapagliflozin has not been studied yet. In the current study, we propose that the potential vasodilatory effect of dapagliflozin may play a role in contributing to its beneficial effects on blood pressure. This discovery holds notable implications as it could potentially lead to blood pressure reductions and enhanced cardiovascular outcomes. Consequently, this may contribute to reducing cardiovascular morbidity and mortality. 

In this study, we observed the vasorelaxant effect of dapagliflozin in rat coronary arteries contracted with high K^+^ and U-46619 (Figure 2A,B). Using these vasoconstrictor agents, we investigated whether dapagliflozin could induce vasodilation in constricted arteries through direct membrane depolarization and/or agonist stimulation. In either case, we found no notable distinction in the vasodilatory response induced by dapagliflozin, implying that dapagliflozin induces relaxation regardless of the type of stimulation. Phlorizin is a non-specific SGLT1 and SGLT2 inhibitor and the first SGLT inhibitor reported to lower blood glucose and normalize insulin sensitivity [14]. In this study, we investigated whether dapagliflozin-induced vasodilation resulted from the inhibition of SGLT2. If the effect of dapagliflozin is attributed to SGLT2 inhibition, similar outcomes should be observed with other SGLT2 inhibitors. Therefore, we also employed a different inhibitor to corroborate our findings. We confirmed that phlorizin did not induce vasodilation in rat coronary arteries (Figure 3B). These findings propose that the vasodilation triggered by dapagliflozin is not a consequence of SGLT2 inhibition but rather an intrinsic characteristic of this compound, accountable for its diverse vascular effects.

Vasodilation occurs through the relaxation of smooth muscle cells within blood vessel walls. This relaxation can result from the removal of a contractile stimulus or the direct influence of vasodilatory agents [15]. The luminal surface of vessels is covered by a monolayer of endothelial cells comprising the vascular endothelium [16,17]. When the endothelium is exposed to different stimuli, it releases substances that induce vasodilation, including nitric oxide (NO), prostacyclin (prostaglandin I_2_; PGI_2_), and endothelium-derived hyperpolarizing factor (EDHF) [18,19,20,21]. In this research, we assessed whether dapagliflozin induces vasodilation by acting through the endothelium. We found that even after the removal of the endothelium, the vasodilatory effect was consistently maintained in rat coronary arteries (Figure 4). To confirm that dapagliflozin-induced relaxation is independent of the endothelium, we used L-NNA, ODQ, and indomethacin. Nitric oxide is produced by eNOS within the endothelium, diffusing subsequently into smooth muscle cells and initiating sGC activation to elevate intracellular cyclic guanosine monophosphate (cGMP) levels, thereby inducing relaxation [22]. The eNOS inhibitor L-NNA was used to confirm the endothelium-independent vasodilatory effect of dapagliflozin (Figure 5A). Treatment with L-NNA had no effect on dapagliflozin-induced vasodilation in coronary arteries. We determined that the sGC inhibitor, ODQ, did not alter the vasodilation induced by dapagliflozin (Figure 5B). Our results indicate that the NO/cGMP pathway is not involved in dapagliflozin-induced vascular relaxation. The results align with a previous investigation conducted by Ahasanul Hasan et al. which demonstrated dapagliflozin-induced endothelium-independent vasodilation in rat mesenteric arteries [23]. They also found that treatment with L-NNA and endothelial removal did not affect dapagliflozin-induced vasodilation.

Furthermore, we investigated whether the vasodilatory effect of dapagliflozin is affected by another vasoactive substance, PGI_2_, which is generated by COX [24,25]. The binding of PGI_2_ to the prostaglandin I_2_ receptor (IP receptor) on smooth muscle cells triggers the activation of adenylate cyclase, leading to the production of cyclic adenosine monophosphate (cAMP) [26]. Then, cAMP activates protein kinase A, leading to the relaxation of smooth muscle in the same way as NO [27]. We observed that the non-selective COX inhibitor indomethacin did not affect dapagliflozin-induced vasodilation. These findings confirmed that the vasodilatory effect induced by dapagliflozin occurs through an endothelium-independent pathway. After we defined that the endothelium is not involved in dapagliflozin-induced vasodilation, we investigated whether dapagliflozin directly acts on smooth muscle cells to induce relaxation [28,29]. The relaxation of vascular smooth muscle initiates with a decrease in intracellular Ca^2+^, which results from a reduction in extracellular Ca^2+^ influx or the re-uptake of Ca^2+^ to the sarcoplasmic reticulum (SR) [15]. Arterial smooth muscle and endothelial cells contain several K^+^ channels involved in membrane potential and vascular tone regulation [30,31]. The activation of K^+^ channels induces an efflux of K^+^, leading to membrane hyperpolarization. Consequently, this results in the closure of voltage-dependent Ca^2+^ channels, blocking extracellular Ca^2+^ influx and inducing the relaxation of smooth muscle cells [32]. In this study, we examined whether dapagliflozin-induced vasodilation involves K^+^ channel activation. A non-selective K^+^ channel inhibitor, TEA, had no effect on dapagliflozin-induced vasodilation (Figure 6A). To verify these results, we incubated arteries with various blockers for different types of K^+^ channels. We found that treatment with 4-AP, glibenclamide, and BaCl_2_ did not alter the effect of dapagliflozin, suggesting that K^+^ channels, including the voltage-gated potassium channel blocker, ATP-sensitive K^+^ channel blocker, and inward rectifier K^+^ channel, were not involved in the vasodilatory response induced by dapagliflozin (Figure 6B–D). Our data do not align with previous studies, as earlier research indicated the involvement of K_V_ channels in the vascular dilation response to dapagliflozin in the mesenteric artery [23]. We assume that these differences may arise from differences in the type of animals or type of blood vessels.

Since we confirmed that endothelial cells and K^+^ channels are not involved in the vasodilation induced by dapagliflozin, we investigated whether dapagliflozin directly inhibits an increase in intracellular Ca^2+^ levels in smooth muscle cells. Since a Ca^2+^ channel blockade did not induce arterial contraction, we were unable to test the relaxing effect of dapagliflozin in the presence of Ca^2+^ channel blockers. Therefore, we employed alternative methods to test the effects of dapagliflozin. We incubated coronary arteries in a Ca^2+^-free K-H solution containing CPA, which depletes intracellular Ca^2+^. Then, the solution was switched to one containing 70 mM of K^+^ to enable the opening of voltage-dependent Ca^2+^ channels. The cumulative addition of Ca^2+^ elicited a contraction response in coronary arteries which was attenuated by the administration of dapagliflozin (Figure 7B). Based on these results, we assumed that dapagliflozin treatment leads to the inhibition of extracellular Ca^2+^ influx. Our results are in accordance with a previous study which showed that dapagliflozin attenuates diabetic cardiomyopathy through a reduction in intracellular Ca^2+^ overload [33]. Although a direct structural relationship between dapagliflozin and Ca^2+^ channels was not suggested in the current study, these data suggest the possibility that dapagliflozin may affect Ca^2+^ channels.

When the intracellular Ca^2+^ concentration increases in vascular smooth muscle cells, myosin light chain kinase (MLCK), which phosphorylates MLC_20_, is activated. The phosphorylation and dephosphorylation of MLC_20_ are regarded as significant events in the regulation of smooth muscle contraction [34]. Therefore, molecules regulating MLC_20_ phosphorylation are considered key determinants of muscle contraction. Furthermore, Ca^2+^ binds with calmodulin, forming the Ca^2+^–calmodulin complex, which activates the MLCK responsible for phosphorylating the serine 19 (Ser^19^) of MLC_20_. Phosphorylated MLC_20_ triggers the activation of myosin ATPase, resulting in muscle contraction [35]. Thus, the phosphorylation of MLC_20_ is vital for smooth muscle contraction. In contrast, a reduction in intracellular Ca^2+^ enables an opposing response. Given the observed reduction in contractions induced by Ca^2+^ in coronary arteries due to dapagliflozin, we proceeded to investigate whether dapagliflozin affects the phosphorylation level of MLC_20_ in vascular smooth muscle cells. We discovered that treatment with U-46619 leads to an increase in the phosphorylation level of MLC_20_ in AoSMCs. The combined treatment with U-46619 and dapagliflozin significantly reduced the phosphorylation level of MLC_20_ compared to U-46619 treatment alone (Figure 8A). Even though we did not directly measure changes in the intracellular Ca^2+^ concentration induced by dapagliflozin, the above findings suggest that dapagliflozin reduces MLC_20_ phosphorylation, ultimately leading to vascular relaxation [36,37,38]

Our findings support the potential multifaceted cardiovascular benefits of dapagliflozin, including a decrease in vascular tone, the attenuation of vascular inflammation and atherosclerosis, the modulation of sympathetic nerve activity, the regulation of natriuretic peptides, and the inhibition of sodium–hydrogen exchange [1]. This suggests the possibility that dapagliflozin may have additional molecular targets beyond blocking SGLT2 in the renal tubules, which could result in beneficial cardiovascular effects across various cells, tissues, and organ systems. Although dapagliflozin has a significant vasodilator effect, there has not yet been sufficient research regarding its improved cardiovascular function. Nevertheless, further investigations are needed to investigate whether chronic dapagliflozin administration triggers vasodilation in resistance arteries and subsequently decrease systemic blood pressure in vivo.

In this study, we discovered that dapagliflozin induces concentration-dependent vascular relaxation in rat coronary arteries which is associated with an endothelium-independent mechanism. Furthermore, we also found that the NO/cGMP pathway and prostacyclin are not involved in the vasodilatory effect induced by dapagliflozin. The inhibition of extracellular Ca^2+^ influx was found to be associated with the vasodilation induced by dapagliflozin. Our data contribute to providing basic knowledge to build the therapeutic potential of dapagliflozin as an anti-hypertension treatment.

## 4. Materials and Methods

### 4.1. Animals and Tissue Preparation

All experiments were performed according to the Guide for the Care and Use of Laboratory Animals published by the US National Institutes of Health (NIH publication No. 85–23, 2011) and were approved by the Ethics Committee and the Institutional Animal Care and Use Committee of Yonsei University College of Medicine (approval number: 2022-0164). In total, 68 rats were used in this study. In this experiment, 13-week-old male Sprague Dawley rats were used. After each rat was sacrificed, the heart was excised, and the coronary arteries were quickly dissected. The left descending coronary arteries were cut into segments 2–3 mm long and placed in an ice-cold Krebs–Henseleit (K-H) solution (composition mmol/L: NaCl, 119; KCl, 4.6; MgSO_4_, 1.2; KH_2_PO_4_, 1.2; CaCl_2_, 2.5; NaHCO_3_, 25; and glucose, 11.1) that was bubbled with 95% O_2_ and 5% CO_2_. Adipose and connective tissue were removed from the coronary arteries using a microscope (model SZ-40, Olympus, Tokyo, Japan). In the endothelium-denuded experiments, the endothelium was removed by perfusing the vessels with 0.1% Triton X-100 for 10 s, followed by an additional 10 s of perfusion with K-H solution [39]. Endothelium denudation was confirmed via the absence of relaxation induced by acetylcholine (ACh, 10 μM)

### 4.2. Measurement of Isometric Tension in Coronary Arteries

The coronary artery segments were mounted in a wire myograph (model 620 M, Danish Myotechnology, Aarhus, Denmark) for measuring isometric tension. Arterial rings were bathed in a 37 °C K-H solution aerated with 95% O_2_ and 5% CO_2_. The arteries were equilibrated for 20 min and stretched to their optimal resting tension (2 mN). The contractility of the vessels was evaluated by incubating the arteries three times in a high-K^+^ solution (70 mM) (composition mmol/L: NaCl, 53.6; KCl, 70; MgSO_4_, 1.2; KH_2_PO_2_, 1.4; CaCl_2_, 2.5; NaHCO_3_, 25; and glucose, 11.1). The response was recorded by stabilizing the vessel by contracting the arteries using high K^+^ (70 mM) or U-46619, followed by the cumulative addition of dapagliflozin (500 μM) or a vehicle (dimethyl sulfoxide: DMSO, 0.0006–0.284%). In addition, to investigate the relaxation effect induced by other SGLT2 inhibitors, the coronary arteries were pre-constricted using U-46619 (500 nM) and then phlorizin (10 μM) was administrated. To investigate the mechanism of vascular relaxation induced by dapagliflozin, *N*^ω^-Nitro-l-arginine (L-NNA), indomethacin, 1*H*-(1,2,4)oxadiazolo[4,3-a] quinoxalin-1-one (ODQ), tetraethylammonium chloride (TEA), barium chloride (BaCl_2_), glibenclamide, and 4-aminopyridine (4-AP) were pre-treated for 20 min, and the relaxation response to dapagliflozin (500 μM) in a U-46619 (500 nM)-induced contraction was recorded. To determine the involvement of Ca^2+^ influx in dapagliflozin-induced vasodilation, the K-H solution was replaced with Ca^2+^-free K-H (composition mmol/L: NaCl, 119; KCl, 4.6; MgSO_4_, 1.2; KH_2_PO_4_, 1.2; NaHCO_3_, 25; glucose, 11.1; ethylene glycol bis (2-aminoethyl) -*N,N,N’,N’*- tetraacetic acid (EGTA), 1) solution containing high K^+^ (70 mM) and a sarcoplasmic reticulum Ca^2+^-ATPase (SERCA) inhibitor, cyclopiazonic acid (CPA, 5 μM), for emptying the intracellular Ca^2+^; then, CaCl_2_ was cumulatively added. After a 20 min pre-treatment with dapagliflozin (500 μM) in the same arteries, the changes in contractility due to another cumulative addition of CaCl_2_ were compared to those observed before the dapagliflozin pre-treatment. The CaCl_2_-induced contraction was calculated as the percentage of the maximum contraction recorded from the KCl contraction.

### 4.3. Cell Culture

Human aortic smooth muscle cells (AoSMCs) were purchased from Lonza (Walkersville, MD, USA). The AoSMCs were cultured in smooth muscle cell basal media supplemented with various growth factors (SmGM-2, including insulin, fibroblast growth factor, epidermal growth factor, and 2% serum). The cell cultures were maintained in a cell incubator at 37 °C with a humidified atmosphere containing 5% CO_2_. Between four and six passages of cells were used for the experiments. Prior to experimentation, the cultured AoSMCs were serum-starved for 18 h in a smooth muscle cell basal medium (smBM^TM^) without FBS.

### 4.4. Measurement of MLC_20_ Phosphorylation Level (Western Blot Analysis)

The cultured AoSMCs were treated with a vehicle (0.1% DMSO), U-46619 (100 nM), or U-46619 (100 nM) with dapagliflozin (50 μM) and then homogenized in an ice-cold lysis buffer as described previously [40]. Cell lysates were centrifuged at 15,000 rpm for 20 min at 4 °C, supernatants were collected, and protein concentrations were normalized using the bicinchoninic acid (BCA) method. Approximately 65 μg of protein for each sample was processed for Western blotting. After resolving protein on a 15% SDS-PAGE gel, protein was transferred onto nitrocellulose (NC) membrane using a transfer method [41,42]. The NC membranes were blocked with 5% skim milk in TBST (tris-buffered saline with 0.1% Tween 20). Blots were incubated with phosphorylated 20 kDa myosin light chain (MLC_20_) antibody and total MLC_20_ antibody (Cell Signaling, Boston, MA, USA) at 1:500 dilution overnight at 4 °C. The blots were then washed three times and incubated with mouse anti-rabbit lgG-HRP secondary antibody (1:1000 dilution) for 1 h at room temperature. At the end of the secondary antibody incubation, NC membranes were washed three times. The membranes were developed using an enhanced chemiluminescence (ECL; Cytiva, Amersham, UK) detection solution, and protein bands were imaged using an AGFA CP 1000. The blots were stripped and then reprobed with the β-actin antibody (1:3000 dilution; Santa Cruz Biotechnology, Santa Cruz, CA, USA) to verify equal loading between the samples.

### 4.5. Chemicals and Reagents

U-46619, ACh, *N*^ω^-Nitro-l-arginine (L-NNA), 1*H*-(1,2,4)oxadiazolo[4,3-a]quinoxalin-1-one (ODQ), Tetraethylammonium chloride (TEA), BaCl_2_, 4-Aminopyridine (4-AP), dimethyl sulfoxide (DMSO), ethylene glycol bis(2-aminoethyl) tetraacetic acid (EGTA), and phlorizin were purchased from Sigma-Aldrich (St. Louis, MO, USA). Indomethacin was obtained from Calbiochem (Darmstadt, Germany). Glibenclamide was purchased from Tocris Bioscience (Avonmouth, Bristol, UK). Dapagliflozin was purchased from MCE (Monmouth Junction, NJ, USA). Cyclopiazonic acid (CPA) was obtained from Enso life Sciences (Farmingdale, NY, USA). The dapagliflozin exhibited a purity of 99.87%, and the phlorizin showed a purity of ≥99%. All other chemicals used in this study were of analytical reagent grade.

### 4.6. Statistical Analysis

Mean values ± standard deviations are presented. Normal distribution of the data was confirmed using the Shapiro–Wilk test. One-way or two-way ANOVAs were used to compare the groups and were followed by multiple-comparison testing using Bonferroni’s post hoc test. In all experiments assessing vascular tension, the “*n*-values” denote the number of arteries derived from distinct animals. The “*n*-value” indicates the number of independent experiments in the Western blot analysis. Statistical analyses were performed using GraphPad Prism Version 10.0.0 (GraphPad Software Inc., San Diego, CA, USA).

## Data Availability

Data are contained within the article.
